# Charters in Patient‐Engaged Research: A Scoping Review of Current Evidence

**DOI:** 10.1002/puh2.70179

**Published:** 2025-12-22

**Authors:** Jenny Martínez, Catherine Verrier Piersol, Richard W. Hass, Felicia Chew, Amy Cunningham, Sharon Larson

**Affiliations:** ^1^ College of Population Health Thomas Jefferson University Philadelphia Pennsylvania USA; ^2^ Department of Occupational Therapy Jefferson College of Rehabilitation Sciences Thomas Jefferson University Philadelphia Pennsylvania USA; ^3^ Department of Family and Community Medicine Thomas Jefferson University Philadelphia Pennsylvania USA

**Keywords:** comparative effectiveness research, methodology, patient engagement, systematic review, translational research

## Abstract

**Aims:**

Research study charters facilitate shared governance and power sharing with research partners when developed collaboratively and early on in a study. Study charters are negotiated and developed on a study‐by‐study basis, offering insight into study‐specific dynamics between research partners and investigators, as well as the factors that each identify as important for their collaboration. This review aimed to map and synthesize existing evidence on the development, structure, and implementation of research study charters used in patient‐engaged research.

**Methods:**

We searched peer‐reviewed literature published worldwide in English between January 2019 and January 2025.

**Results:**

Our final sample consisted of 17 citations, including 5 journal articles and 12 charters available on Patient‐Centered Outcomes Research Institute's (PCORI's) Engagement Tool and Resource Repository. Research study charters varied in their development, structure, and content.

**Conclusions:**

We identified research study charters that varied in quality, detail, and methods. Valid, systematic, and inclusive study charters that are developed with research partners and reflect a diversity of perspectives can improve governance and engagement in research partnerships. Their adoption may enhance the quality and inclusivity of patient‐centered research and represent an important area for future investigation.

## 1 Introduction

Systematically and purposefully engaging community members (e.g., patients, caregivers, practitioners, policy makers, and payers) as research partners in study development and conduct is crucial for science that is actionable, timely, and directly relevant to communities. Specifically, patient accounts can provide valuable insight into the inequities experienced by minoritized populations and their intersection with disease, medical mistrust, the longstanding effects of scientific racism and discrimination, and the systemic barriers to health and well‐being. An in‐depth understanding of medical conditions, challenges in everyday life, valued goals, and needs not met by available treatments may also be gained, thereby improving the relevance of research findings to patients from all backgrounds [1, 2, 3]. It is essential that research include participants who reflect the local communities and a wide variety of lived experiences, living environments, and individual characteristics. Prior studies have shown that such engagement improves the relevance of research, builds trust, and enhances outcomes [4, 5, 6].

As advocates, funders, and investigators look to expand research that involves patients and other community members as research partners, best practices for implementing high‐quality engagement programs are needed. Such strategies are especially timely given low knowledge of—and participation in—clinical trials by various populations, including minoritized communities [7, 8, 9]. Even after understanding the benefits of engaging patients from minoritized populations as research partners, investigators may lack the knowledge of specific practices to do so. Furthermore, research partners may feel powerless to influence study decisions, unfamiliar with the project or related topics, disempowered in relationships with researchers, stressed by excessive demands on their time, and inadequately compensated for their effort.

Establishing a research study charter, referred to as *study charter(s)* in this article, is one strategy for investigators to develop and implement meaningful and authentic relationships with research partners. Study charters facilitate shared governance and power sharing with research partners when developed collaboratively and early on in a study [10]. Through this process, investigators and their research partners identify common guidelines, policies, roles, responsibilities, and governance structures that serve as the foundation for each study partnership. Study charters are negotiated and developed on a study‐by‐study basis, offering insight into study‐specific dynamics between patient partners and investigators and the factors each identify as important for their research collaboration.

Study charters offer a unique examination of research collaborations in ways that other materials (e.g., training manuals, literature reviews) cannot. To this end, we conducted a scoping review of research study charters as a first step to understanding current practices for engagement. This article presents a descriptive overview of our findings and identifies areas for future research to expand authentic and meaningful engagement of patients and other community members as research partners. Unlike training manuals or literature reviews, study charters formalize expectations, roles, and governance structures, providing a concrete framework for collaboration and accountability.

### 1.1 Research Question

This scoping review centers on the study charter, a well‐established method for helping research partners and investigators jointly establish guidelines and policies, delineate roles, and clarify study governance structures at the outset of a project [10]. Given the importance of the patient voice in research, this scoping review is focused on the experiences of patient research partners. In accordance with recent definitions in patient‐centered research, this study defines *patient research partners* as individuals that bring lived experience with a condition (i.e., patient, survivor, and informal care partner of someone with the condition) that collaborate with investigators as respected experts with decision‐making authority [3, 11, 12]. Specifically, this scoping review aimed to map and synthesize existing evidence on the development, structure, and implementation of research study charters used in patient‐engaged research.

## 2 Methods

This scoping review uses the seminal methodological framework developed by Arksey and O'Malley [13] that makes use of a staged process that includes identifying the research question, identifying relevant studies, selecting studies, charting the data, and summarizing and reporting the results as part of its methodology [13, 14, 15].

Scoping reviews are an increasingly popular approach for synthesizing the diverse body of literature on a given topic with a broader perspective than the systematic review, given their ability to map the extent, range, and nature of research activity in an area that has not yet been extensively reviewed. Scoping reviews also use more inclusive and efficient ways of gathering evidence. For example, they may include a wide range of study designs and methods and omit the critical appraisal of articles [15]. Moreover, the scoping review streamlines methods common across other approaches for synthesizing evidence, leading to trustworthy results in a faster timeframe [15]. Finally, scoping reviews are completed by an individual researcher experienced in the technique and have been shown to provide results similar in breadth and depth to a systematic review [14]. Given these characteristics and estimates that systematic reviews can take up to 2 years to complete with necessary expertise from a multi‐person research team, the scoping review is an attractive strategy for examining emergent topics and providing an overview of current literature [16].

### 2.1 Patient Advisors

Patient Advisors were convened to guide this scoping review and increase project transparency, trustworthiness, and patient‐centeredness. The advisors included two clinician‐researchers with lived experience as care partners and patient research partners. Their dual expertise as clinicians and patient partners provided valuable insights into the lived experiences of patients and caregivers, while also being intimately acquainted with research practices and emergent needs for patient engagement.

Patient Advisors convened three times at a convenient time and venue for the members—twice virtually and once in person. The members guided the scoping review search strategy, contributed to data analysis and interpretation, and provided feedback on questions and issues that arose throughout the process. Both Patient Advisors received a $100 stipend for their participation in recognition of their valuable expertise and role.

### 2.2 Data Sources and Search Terms

Study charters were the focus of this scoping review, as they play a crucial role in patient‐engaged research by formalizing shared expectations, roles, and governance structures between investigators and patient partners. By collaboratively developing charters, research teams foster transparency, trust, and accountability, ensuring that patient perspectives are meaningfully integrated into all stages of the research process. Charters are also publicly available via publication in scientific publications and in resources from funders such as Patient‐Centered Outcomes Research Institute (PCORI) as examples of strategies for community‐engaged research.

The scoping review utilized a comprehensive search strategy that combined major terms, such as “patient partner, stakeholder engagement, patient engagement, research, clinical trial, and charter,” as presented in Table 1. Patient Advisors provided further insight into the best terms to utilize and helped shape the search strategy. For example, the members recommended a focused search on “charters” instead of “guidelines” or “procedures” to remain focused on study guidelines instead of patient care guidelines. They also supported the use of a focused search centered on “charters” because these are commonly used features of governance structures and committees. Patient Advisors also recommended search terms that reflected the important role of caregivers in the patient experience.

**TABLE 1:** Scoping review search terms.

Search terms	Search string
Patient	((patient) OR (stakeholder) OR (caregiver) OR (care partner) OR (community member))
Research partner	((research partner) OR (patient research partner)) ((advisory) OR (committee) OR (council) OR (panel)) ((research OR study OR science OR clinical trial))
Charter	((charter))

### 2.3 Search Strategy

The search was conducted in January 2025 and was limited to articles published in English between January 2019 and January 2025. This timeframe was selected to capture the most recent developments and discussions about inpatient‐centered research since PCORI's funding in 2013 [17]. The search utilized select main databases, including PubMed, ProQuest Social Sciences, and CINAHL [15, 18, 19]. PCORI's Engagement Tool and Resource Repository was also manually searched for eligible documents [20]. The year 2019 was selected to capture recent developments in patient‐centered research following PCORI's funding initiatives. Searches required at least two terms, including “charter” and one of “patient partner,” “stakeholder engagement,” or “patient engagement.” We acknowledge that terms like “memorandum of agreement” or “memorandum of understanding” may also be relevant and suggest future reviews to consider terms such as these.

Articles were included if they described patients who were engaged as partners, not just participants, and if the article described patient involvement in developing expectations about aspects such as study roles, responsibilities, and expectations. The focus on these types of articles was intentional and designed to reflect studies that reflected true engagement of patients, not just tokenistic involvement or requests for input without decision‐making power [12]. Study charters play a crucial role in patient‐engaged research by formalizing shared expectations, roles, and governance structures between investigators and patient partners. By collaboratively developing charters, research teams foster transparency, trust, and accountability, ensuring that patient perspectives are meaningfully integrated into all stages of the research process. Given the scope of this project, we focused on collaboratively developed charters to examine shared governance and partnership dynamics. However, we recognize that non‐collaborative charters may offer insights, and we recommend future research explore this distinction. Similarly, relevant documents within the timeframe published on PCORI's Engagement Tool and Resource Repository were included [20]. Peer‐reviewed journal articles published in a language other than English, conference abstracts, and letters to the editor were excluded due to the limited detail they provided on the topic.

### 2.4 Screening and Analysis

**FIGURE 1:**
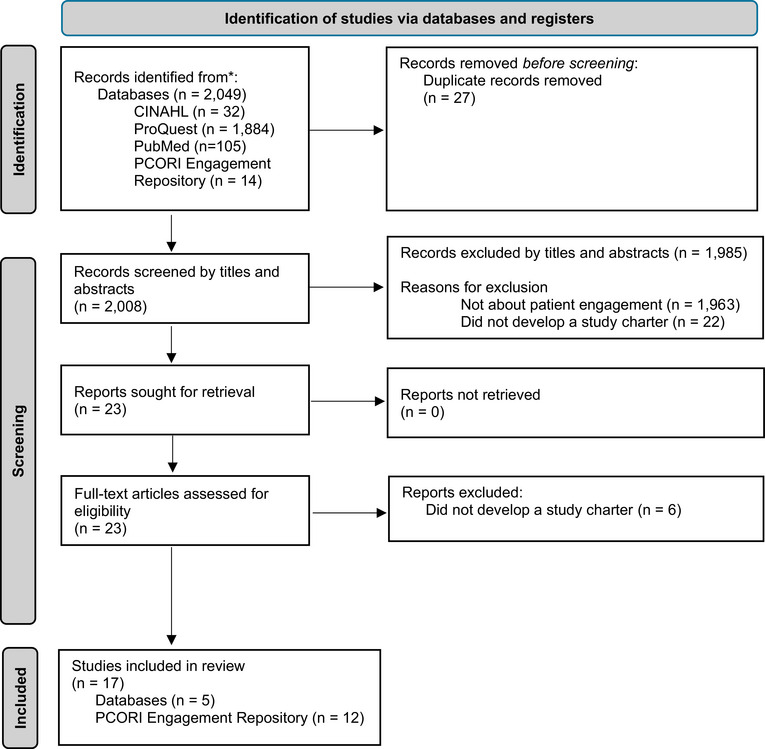
Search and inclusion flow diagram. PCORI, Patient‐Centered Outcomes Research Institute.

The process of screening and finalizing the sample for analysis is summarized in Figure 1 [21]. First, abstracts were compiled and duplicates removed. The investigator (blinded for review) reviewed the titles and then abstracts of retrieved publications for possible inclusion with input from the Patient Advisors. All potential articles for the final sample were reviewed by the investigator and Patient Advisors. The final sample was identified through discussion and deliberation between the Patient Advisors and investigator. Disagreements were discussed with three other project collaborators (blinded for review).

Data extraction and analysis were completed by the investigator in collaboration with the Patient Advisors. A standardized form (Table 2) was used to capture information about the patient partners and investigators represented and the factors identified by patients and investigators as important for their collaborations that were reported in each article or document. Information extracted included (a) study year and publication, (b) description of the materials reviewed, (c) individuals who participated in developing the study charter, (d) steps taken to develop the study charter, and (e) components of the study charter. Study charter components were also reviewed using PCORI's Foundational Expectations for Partnerships in Research to facilitate comparison and description across documents. The PCORI Foundational Expectations for Partnerships in Research were chosen as a common framework given their role as six well‐known building blocks to strengthen engagement with patients and other research partners [12]. Further, the use of these widely recognized standards for patient and stakeholder engagement in research permits a consistent basis for comparison across charters.

**TABLE 2:** Study charters identified in the literature (*N* = 17).

Reference	Description of materials	Who participated in developing the study charter	Steps taken to develop the study charter	PCORI foundational expectations for partnerships in research addressed	Components of study charter
*Journal articles (n = 5)*					
[22]	This journal article presents guidelines for data monitoring committees in future studies. A sample charter is not provided	The journal article states that the sponsor, the trial's executive committee, the statistical data analysis center, and the data monitoring committee (DMC) members would take part in developing a study charter	The journal article recommends that an initial draft should be developed by the sponsor in collaboration with the trial's executive committee and substantive input from the DMC. It is also recommended that the DMC should provide targeted feedback on the initial draft after reviewing the charter, the protocol, and the statistical analysis plan	Diversity and representation Early and ongoing engagement Build capacity to work as a team Meaningful inclusion of partners in decision‐making	Rationale for use of a DMC Broad goals, roles, responsibilities, and operational structure of the DMC Decision‐making process of the DMC DMC composition, including the number and expertise areas of its members Scheduled data transfers from the trial's data management group to the Statistical Data Analysis Center Format and frequency of meetings Flow of communication between DMC and other relevant groups (e.g., other committees and sponsors)
[23]	This protocol paper outlines a process for public and patient involvement (PPI) in the organizational science and management principles side of healthcare. A sample charter is not provided	The journal article states that charter development will include investigators and a PPI panel consisting of patients and caregivers with recent experience in the Irish healthcare system	The journal article proposes using three workshop‐style meetings to design the charter, with each meeting lasting approximately two hours. Meeting one will build rapport and clarify roles. PPI members will also identify what they would like to gain from involvement in the panel; the charter is expected to be approved by the end of the third meeting	Diversity and representation Early and ongoing engagement Build capacity to work as a team Ongoing review and assessment of engagement	Although no specific charter components are identified, the authors state they will discuss a range of topics with the PPI during their initial meetings, including: – Diverse PPI membership – Role delineation – PPI member interests for learning and gains from participating – Identify investigator and PPI research priorities – How the impact of PPT should be assessed and what should be evaluated (e.g., experiences of engagement and perspectives on various aspects of the study)
[24]	This journal article describes the integration of a data monitoring committee in a retrospective, joint industry‐sponsored thyroid cancer study. A template charter is provided as an appendix	The journal article states that charter development included sponsors of an active thyroid cancer trial, members of a professional organization comprised of thyroid cancer specialists, and Data Committee Members (DMC). This information is not included in the template charter	The journal article states that sponsors held discussions internally and collaborated with the professional organization of thyroid specialists, leading to an initial draft. Next, an initial draft was reviewed by all sponsors participating in the active trial. The sponsors also held a kick‐off meeting for DMC members to discuss the charter. Lastly, the charter was finalized post agreement between all sponsors, the DMC chair, and the contract research organization	Early and ongoing engagement	When a DMC is indicated Establishing a DMC charter DMC membership and composition DMC roles and responsibilities DMC meeting structure Expected DMC products (e.g., formal recommendations) and flow of communication with relevant groups (e.g., other committees and sponsors) Signature line for each partner
[10]	This journal article presents the Stakeholder‐Centric Engagement Charter, a charter developed within a PCORI‐funded large pragmatic trial investigating treatment approaches for dementia. The full‐text charter is provided as an appendix	The journal article states that charter development included study investigators and an 18‐member advisory committee that included representation from communities such as patient advocates, caregivers, practitioners, policy makers, and health system leaders. This information is not included in the charter	The journal article states that the investigators created an initial outline of the charter. The initial outline was discussed and reviewed by the investigator and advisory committee during the study kick‐off meeting, leading to a working draft. Next, the working draft underwent targeted review by patient and caregiver advisory committee members. Finally, the working draft was then reviewed by the entire advisory committee and approved	Diversity and representation Early and ongoing engagement Dedicated funds for engagement and partner compensation Build capacity to work as a team Meaningful inclusion of partners in decision‐making Ongoing review and assessment of engagement	Advisory committee overview, composition, and membership Advisory committee onboarding and orientation procedures Advisory committee compensation Advisory committee expectations for participation and contributing to an inclusive environment Investigator expectations for participation and contributing to an inclusive environment Participation overview and procedures (e.g., meetings and regular evaluation) Conflict resolution procedures Stepping down or removal from the advisory committee Advisory committee‐initiated opportunities to disseminate research findings Signature lines for each partner
[23]	This journal article presents the Canadian Cancer Trial Stakeholder Charter, a charter used to operationalize the Canadianized version of the Clinical Trials Transformation Initiative in cancer. The charter is included in the body of the article	The journal article states that charter development included a working group of scientists/clinicians, patient group and advocacy group representatives, cooperative academic clinical trial oncology groups, representatives from health agencies, research networks/consortia, industry partners, contract research organizations, and a non‐profit colorectal cancer patient organization. The members are listed in an appendix	The journal article describes a series of working group sessions leading to an initial draft that was circulated to various groups, including patient groups and trial sponsors. Next, the updated draft was discussed during the annual conference held by the non‐profit colorectal cancer patient organization. Lastly, the working group convened to finalize the charter	Early and ongoing engagement Build capacity to work as a team	Making patient centricity a norm in clinical trials Supporting education, training, and development of patient group members Collaborating with patient groups as equal and independent partners Adhering to transparency and accountability Maximizing the potential to collect and utilize real‐world evidence and real‐world data No signature line
*Standalone study charters available on the PCORI engagement repository (n = 12)*					
[25]	The charter is available on PCORI's Engagement Tool and Resource Repository	The charter does not describe who participated in its development but lists groups that are represented on the committee	The charter states that roles, and decision‐making authority of the committee will be established collaboratively during the first set of meetings. Members will also describe what they hope to achieve or learn through their participation	Diversity and representation Early and ongoing engagement Build capacity to work as a team Ongoing review and assessment of engagement	Membership of the Study Advisory Committee Committee operations Committee responsibilities
[26]	The charter is available on PCORI's Engagement Tool and Resource Repository	The charter does not describe who participated in its development but lists groups that are represented on the committee	The charter states that roles, and decision‐making authority of the committee will be established collaboratively during the first set of meetings. Members will also describe what they hope to achieve or learn through their participation	Diversity and representation Early and ongoing engagement Build capacity to work as a team	Membership of the Implementation Monitoring Committee Committee operations Committee responsibilities
[27]	The charter is available on PCORI's Engagement Tool and Resource Repository	The charter does not describe who participated in its development, but the PCORI page for the project mentions that research partners helped create and finalize the policies and procedures	The charter does not describe how it was developed	Diversity and representation Build capacity to work as a team	Purpose of the Governing Board Governing Board membership and leadership Responsibilities of the Governing Board Governing Board Meetings Voting Rules
[28]	The charter is available on PCORI's Engagement Tool and Resource Repository	The charter does not describe who participated in its development, but the PCORI page for the project mentions that the Governing Board approved policies and procedures	The charter does not describe how it was developed, but the PCORI page for the project mentions that the Governing Board approved policies and procedures	Early and ongoing engagement Build capacity to work as a team	Study structure Responsibilities of the Engagement Committee Study background Committee operations (e.g., structure, elections, and procedures) Signature of Committee co‐chairs on behalf of the Committee
[29]	The charter is available on PCORI's Engagement Tool and Resource Repository	The charter does not describe who participated in its development, but the PCORI page for the project mentions that the Governing Board approved policies and procedures	The charter does not describe how it was developed, but the PCORI page for the project mentions that the Governing Board approved policies and procedures	Early and ongoing engagement Build capacity to work as a team	Study structure Responsibilities of the Governing Board Study background Governing Board operations (e.g., structure, elections, and procedures) Signature of Governing Board chair on behalf of the committee
[30]	The charter is available on PCORI's Engagement Tool and Resource Repository	The charter does not describe who participated in its development, but the PCORI page for the project mentions that the Governing Board approved policies and procedures	The charter does not describe how it was developed, but the PCORI page for the project mentions that the Governing Board approved policies and procedures	Early and ongoing engagement Build capacity to work as a team	Study structure Responsibilities of the Research Committee Study background Committee operations (e.g., structure, elections, and procedures) Signature of Research Committee co‐chairs on behalf of the committee
[31]	The charter is available on PCORI's Engagement Tool and Resource Repository; no publications about the charter found	The charter does not describe who participated in its development	The charter does not describe how it was developed	Dedicated funds for engagement and partner compensation Build capacity to work as a team	Mission of the Interactive Autism Network Long‐term goals of the Interactive Autism Network Purpose of the Community Advisory Council Structure of the Community Advisory Council
					Meetings of the primary and satellite teams of the Community Advisory Council Compensation of the primary and satellite teams of the Community Advisory Council
[32]	The charter is available on PCORI's Engagement Tool and Resource Repository	The charter does not describe who participated in its development but lists groups that are represented on the committee	The charter does not describe how it was developed	Diversity and representation Early and ongoing engagement Dedicated funds for engagement and partner compensation	Parent Action Committee roles and responsibilities Committee membership Committee requirements and compensation
[33]	The charter is available on PCORI's Engagement Tool and Resource Repository	The charter does not describe who participated in its development but lists the groups represented in the Stakeholder Advisory Board	The charter does not describe how it was initially developed but states that it is intended to evolve with input from members of the study team	Diversity and representation Early and ongoing engagement Dedicated funds for engagement and partner compensation Build capacity to work as a team	Project overview Project funding information Advisory Board composition Advisory Board roles and responsibilities Advisory Board participation expectations and compensation No signature line
[34]	The charter is available on PCORI's Engagement Tool and Resource Repository	The charter does not describe who participated in its development	The charter does not describe how it was developed	Diversity and representation Early and ongoing engagement Dedicated funds for engagement and partner compensation Build capacity to work as a team Meaningful inclusion of partners in decision‐making Ongoing review and assessment of engagement	Background and project objectives Partnership overview Partnership structure Partnership principles Engagement activities Participatory decision‐making Project timeline Communication Plan Amendments to charter Signature of individual members of the partnership
[35]	The charter is available on PCORI's Engagement Tool and Resource Repository	The charter does not describe who participated in its development but lists groups that are represented on the committee	The charter does not describe how it was developed	Diversity and representation Build capacity to work as a team	Purpose of the Equality State Research Network Membership of the Equality State Research Network Responsibilities of the Wyoming Institute for Disabilities Responsibilities of members of the Equality State Research Network Responsibilities of the Network Advisory Council Common agreements (e.g., participation, communication, meetings, ground rules) Signature of individual Equality State Research Network member
[36]	The charter is available on PCORI's Engagement Tool and Resource Repository	The charter does not describe who participated in its development but lists the members of the External Advisory Board	The charter does not describe how it was developed	Diversity and representation Early and ongoing engagement Build capacity to work as a team	Purpose of the External Advisory Board Members of the External Advisory Board Meetings Operations Responsibilities Dissemination Presentations and Publications Signature of individual members of the Board

Abbreviation: PCORI, Patient‐Centered Outcomes Research Institute.

## 3 Results

After a systematic process of reviewing inclusion and exclusion criteria across the initial sample as outlined in Figure 1, 23 peer‐reviewed documents were reviewed at the full‐text phase. Of those 23 documents, 6 were excluded because they did not develop a study charter as part of their study. A final sample of 17 peer‐reviewed documents was obtained, analyzed, and presented in Table 2. The majority of these (*n* = 16) were conducted in the United States, with one study completed in Ireland [37]. The results demonstrate wide variability across study charters, including in structure, quality, and content.

Two types of peer‐reviewed documents were identified across the final sample of 17 documents: *journal articles* and *standalone study charters*. *Journal articles* (*n* = 5) describe full‐text manuscripts about research charters. Within this sample, two journal articles [37, 38] addressed recommendations for future engagement or a study protocol, and three [10, 22, 23] included a full‐text charter or template used in their recent study; two were oncology studies, and one investigated Alzheimer's disease and related dementias. *Standalone study charters* (*n* = 12) describe charter documents published on project‐specific webpages on PCORI's Engagement Tool and Resource Repository [24, 25, 26, 27, 28, 29, 31, 32, 33, 34, 35, 38]. A summary is presented in Table 3.

**TABLE 3:** Summary of scoping review findings: Patient‐Centered Outcomes Research Institute (PCORI) foundational expectations for partnerships in research.

		PCORI foundational expectations for partnerships in research addressed					
Reference	Type of publication	Diversity and representation	Early and ongoing engagement	Dedicated funds for engagement and partner compensation	Build capacity to work as a team	Meaningful inclusion of partners in decision‐making	Ongoing review and assessment of engagement
[22]	Journal article without charter	X	X		X	X	
[23]	Journal article without charter	X	X		X		X
[24]	Journal article with charter		X				
[10]	Journal article with charter	X	X	X	X	X	X
[23]	Journal article with charter		X		X		
[25]	Standalone study charter	X	X	—	X	—	—
[26]	Standalone study charter	X	X	—	X	—	—
[27]	Standalone study charter	X	—	—	X	—	—
[28]	Standalone study charter	—	X	—	X	—	—
[29]	Standalone study charter	—	X	—	X	—	—
[30]	Standalone study charter	—	X	—	X	—	—
[31]	Standalone study charter	—	—	X	X	—	—
[32]	Standalone study charter	X	X	X	—	—	—
[33]	Standalone study charter	X	X	X	X	—	—
[34]	Standalone study charter	X	X	X	X	X	X
[35]	Standalone study charter	X	—	—	X	—	—
[36]	Standalone study charter	X	X		X	—	—

No study charters in the final sample (*N* = 17) described their development. However, all three study charters published in journal articles [10, 22, 23] and three standalone study charters [23, 28, 32] broadly identified constituencies or groups of individuals that would, or had participated, in developing the study charter (e.g., subcommittees, patients, caregivers, and clinicians). All five journal articles addressed involved groups and processes within their study methods [10, 22, 23, 37, 38], and six additional standalone study charters addressed this topic on their PCORI project‐specific webpage [25, 26, 29, 30, 31, 35]. All three study charters published in journal articles and six standalone study charters also included space for approval by signature by each member, or by a representative (e.g., chair or co‐chair), of the partnership [24, 29, 30, 31, 32, 36].

### 3.1 PCORI Foundational Expectations for Partnerships in Research Represented Across Charters

Each document reviewed in the final sample (*N* = 17) was compared to PCORI's Foundational Expectations for Partnerships in Research [12]. This choice facilitated comparison across, and descriptions of, study charters. The results of this analysis are described below, outlined in Table 2, and summarized in Table 3.

#### 3.1.1 Diversity and Representation

This PCORI foundational expectation refers to projects that include research partners that reflect the diversity of those affected by the topic [12]. Two study charters published in journal articles [10, 37] and eight standalone study charters [24, 25, 26, 27, 28, 32, 35, 36] addressed this topic. This information was addressed in all five journal articles [10, 22, 23, 37, 38]. Examples of relevant information included identifying the diverse constituencies involved in the corresponding study, including patient research partners, patient advocates, clinicians, and policy makers. For example, one study charter identified partnering with four groups: caregivers, educators, clinicians, and researchers [36]. Next, the study charter identified names of members, their role in the partnership (e.g., leader and member), and the group they identified with (e.g., caregiver and researcher) [36].

#### 3.1.2 Early and Ongoing Engagement

The PCORI foundational expectation describes the engagement of research partners early and throughout the study's life [12]. All three charters published in journal articles [10, 22, 23] and 10 standalone study charters [24, 25, 27, 28, 29, 31, 32, 36] addressed this topic. Early and ongoing engagement was also included in all five journal articles [10, 22, 23, 37, 38]. Mentions of early and ongoing engagement often included a description of the roles of patient research partners and committee members (e.g., advisors and action boards). Although different for each study, patient research partner and committee member roles included tasks such as helping refine the research question, providing advice on study implementation, and helping disseminate findings. For example, one charter described the role of advisory committee members in advising the research team throughout the project, including advising on study questions, outcomes, protocols, and monitoring [35].

#### 3.1.3 Dedicated Funds for Engagement and Partner Compensation

This PCORI foundational expectation refers to dedicated funds to compensate collaborators, including patient research partners, fairly for their time, expertise, and perspectives [12]. This topic was addressed by one study charter published in a journal article [10], as well as four standalone study charters [27, 28, 34, 36]. Compensation arrangements included (a) reimbursement for approved travel expenses to attend annual in‐person meetings and a $500 honorarium [28], (b) a $200 honorarium for quarterly meetings via phone or in person [27], or a template charter with a discrete area to state payment amount, schedule for payment, and participation requirements [10]. When a compensation amount was not specified, study charters (a) stated that a gift card or similar compensation would be provided [34] or (b) described that all partners would be compensated appropriately [36]. For example, one study described providing advisory committee members with a gift card for their participation [34], whereas another outlined the process for committee members to receive compensation for participation at monthly meetings, including provisions for absences or lateness [10].

#### 3.1.4 Build Capacity to Work as a Team

This PCORI foundational expectation describes the identification of barriers and facilitators to engagement, as well as providing collaborators, including patient research partners, with information and support to work together [12]. Two study charters published in journal articles [10, 22] and all 12 standalone study charters [24, 25, 26, 28, 29, 30, 31, 33, 34, 35, 38] addressed this topic. This topic was also identified by the two journal articles that did not publish an associated study charter [37, 38].

Ways in which capacity to work as a team was addressed included (a) considering member interests for their ongoing learning and development [10, 22, 37], (b) identifying partner roles and expectations for participation [10, 24, 25, 26, 28, 29, 30, 31, 32, 34, 35, 36, 37, 38], (c) identifying expectations for investigators [10, 24], and (d) describing procedures for stepping down, succession, and new member onboarding [10, 26, 29, 30, 31]. In addition, four study charters explicitly named engagement or team values that centered treating all partners, including researchers and patients, as equal and independent contributors [10, 22, 28, 36]. For example, one study charter identified a commitment to reducing power imbalances between members, including patient research partners, through use of various strategies (e.g., use of first names instead of professional titles and incorporation of named partnership principles such as trust and power‐sharing) [10].

#### 3.1.5 Meaningful Inclusion of Partners in Decision‐Making

This PCORI foundational expectation describes efforts to include collaborators, including patient research partners, in decision‐making throughout the project [12]. This expectation was discussed in two study charters published in journal articles [10, 23], one standalone study charter [36], and one journal article that did not publish a charter document [38]. Meaningful inclusion of partners in decision‐making was addressed through descriptions of the flow of communication with other relevant groups [23, 38] and through describing procedures for decision‐making and conflict resolution [10, 36, 38]. For example, Study one charter identified various methods of advisory committee interaction, decision‐making, and participatory decision‐making, along with procedures for resolving disagreements [36].

#### 3.1.6 Ongoing Review and Assessment of Engagement

This PCORI foundational expectation addresses work to gather feedback about the engagement approaches throughout the project from collaborators, including patient research partners [12]. One study charter published in a journal article [10] and one journal article that did not publish a study charter document [37] addressed this topic. Two standalone study charters discussed evaluation [35, 36]. Evaluation efforts included periodic and ongoing feedback requests from partners along with plans to adapt the engagement strategy per feedback received from collaborators, including patient research partners. For example, one study charter identified monitoring the authenticity of engagement using a specific assessment tool during the study period [35].

## 4 Discussion

The findings of this scoping review uncovered a range of approaches and topics for study charters. Study charters were published in peer‐reviewed journals [10, 22, 23], recommended as best practice or described within a protocol paper [37, 38], or published on a funder's website [12]. All study charters addressed at least one of the PCORI's Foundational Expectations for Engagement in Research [12]. Most commonly, study charters addressed two PCORI elements: early and ongoing engagement and building capacity to work as a team. As prior evidence on engagement methods has also identified [1, 30, 37, 38, 39], the least frequently discussed topic in study charters was the process of ongoing review and assessment of engagement. Although study charters are presumed to be important for effective engagement, empirical evidence is limited. We acknowledge this uncertainty and suggest further research to evaluate the impact of charter development.

Somewhat surprisingly, this review found limited available study charters, with varied structure and quality. For example, although five eligible journal articles were identified, only three published a study charter document or template alongside a description of the study charter's use in the respective project [37, 38]. Two additional articles described study charter development and use prospectively but did not include the study charter document [37, 38]. Additionally, study charters did not use a common structure or format. Each study charter differed in content and structure from the next except when multiple research partner committees were part of the same study (e.g., advisory committee and governing board). In these situations, the same study charter document was repurposed [25, 29, 30, 35].

Additionally, this review found that study charter development and use were inconsistently reported. Descriptions of study charters did not describe how they were developed, although they sometimes named the research partners or constituent groups engaged as partners (e.g., patients and clinicians). When the study charter was published as part of a full‐length journal article or on a project webpage, information about study charter development was inconsistently addressed or described. Only about half of the study charters required signature(s) (*n* = 8) [10, 23, 24, 29, 30, 31, 35, 38], with a subset requiring a signature from each member (*n* = 5) [10, 23, 24, 32, 36] and the remaining requiring approval by a representative (e.g., committee chair or co‐chair). Lastly, few if any study charters included an evaluation of engagement, leaving questions about engagement outcomes and the occurrence, content, and quality of an evaluation. Taken together, our findings show that an understanding of the role that research partners truly played in developing joint expectations, approving their use, and adapting the study's engagement strategy is lacking.

This scoping review identified an initial sample (*n* = 2049) that included many studies that reported engaging patient research partners. Consistent with past research, many studies included patients only as participants and not as collaborators. Further still, only a small quantity integrated a study charter as part of their research strategy. Although it is possible that study charter development was conducted and not reported, it is likely that studies have not yet integrated study charter use or development as part of their overall research strategy. When a study charter was used, evaluation of the engagement process was limited, which further decreases the information available about their effectiveness, success, and challenges encountered.

One major challenge to a study charter development and use is the paucity of literature on the topic. As a result, there is little guidance for investigators about setting expectations, building relationships, creating and personalizing the study charter, evaluating effectiveness, and providing ongoing transparent communication of the study charter through the process. In addition, there are few available study charter samples, and those that are published vary in quality and scope, offering limited guidance for investigators. Further, a focus on study results may reduce available space in publications for study charter descriptions or opportunities to publish on charters independently. Still, there is a growing understanding of what themes underlie successful collaboration with patient research partners [12], and a small but growing body of papers that include published study charters that can be adapted or further individualized by investigators [10, 22, 23]. Funder‐specific resources, such as PCORI's Engagement Tool and Resource Repository [20], offer an additional venue to source standalone study charter documents, although these do not offer the contextual information that a journal article would.

### 4.1 Limitations

There are several limitations to this study. First, the quality of the five studies reported in journal articles [10, 22, 23, 37, 38] was not assessed given the scoping review methodology. Assessment was also limited given that many documents were standalone documents rather than full‐text journal articles, which limited the depth of appraisal. As more projects incorporate study charters into their methodology, future reviews should consider evaluating the quality and rigor of these reports.

Next, the review was restricted to study charters published between January 2019 and January 2025, which may have excluded relevant publications released after the search period. The search strategy was limited to English‐language sources, potentially omitting charters developed in other languages or cultural contexts. Additionally, although the search strategy was informed by multiple stakeholder groups, including Patient Advisors, additional relevant documents may have been identified through alternative databases or broader search terms.

Finally, this study also engaged individuals with patient or caregiver experience and research—future work may benefit from including a wider range of patient advisors to enhance representativeness and diversity of perspectives.

### 4.2 Future Perspective

A study charter offers a method for meaningfully engaging research partners in setting joint guidelines and expectations and a venue for ongoing evaluation or discussion about research collaborations. Future work should focus on refining and evaluating study charters. This effort would be supported by additional research on effective strategies to develop and adapt study charters. Moreover, clear guidelines for study charter structure and reporting can assist future researchers in creating common elements for engagement that could facilitate comparison and evaluation. Additional information on how patient research partners and other constituent groups can be authentically engaged in developing study charters is also needed. These efforts will better prepare investigators collaborating with patient research partners to incorporate regular, ongoing, and effective engagement strategies using a collaboratively developed and approved study charter. To best equip future investigators leading patient‐ and community‐engaged research, a consensus on study charters, including their development, structure, and ongoing evaluation, is an important area of future investigation. These findings support the need for defined reporting standards for patient‐engaged research that include charter development and contents. Further research should investigate which aspects of charters are most impactful for engagement outcomes.

## Author Contributions

**Jenny Martínez**: conceptualization, data curation, formal analysis, investigation, methodology, validation, writing – original draft, writing – review and editing. **Catherine Verrier Piersol**: conceptualization, methodology, validation, writing – review and editing. **Richard W. Hass**: conceptualization, methodology, validation, writing – review and editing. **Felicia Chew**: conceptualization, methodology, validation, writing – review and editing. **Amy Cunningham**: conceptualization, methodology, validation, writing – review and editing. **Sharon Larson**: conceptualization, methodology, validation, writing – review and editing.

## Funding

The authors have nothing to report.

## Ethics Statement

This study received ethical approval from the Thomas Jefferson IRB (approval iRISID‐2025‐0154) on March 27, 2025.

## Consent

All participants provided written informed consent, and the study was conducted in accordance with the Declaration of Helsinki.

## Conflicts of Interest

The authors declare no conflicts of interest.

## Data Availability

The authors have nothing to report.
